# Validity of (Ultra-)Short Recordings for Heart Rate Variability Measurements

**DOI:** 10.1371/journal.pone.0138921

**Published:** 2015-09-28

**Authors:** M. Loretto Munoz, Arie van Roon, Harriëtte Riese, Chris Thio, Emma Oostenbroek, Iris Westrik, Eco J. C. de Geus, Ron Gansevoort, Joop Lefrandt, Ilja M. Nolte, Harold Snieder

**Affiliations:** 1 Department of Epidemiology, University of Groningen, University Medical Center Groningen, Groningen, The Netherlands; 2 Department of Vascular Medicine, University of Groningen, University Medical Center Groningen, Groningen, The Netherlands; 3 Interdisciplinary Center Psychopathology and Emotion regulation (ICPE), Department of Psychiatry, University of Groningen, University Medical Center Groningen, Groningen, The Netherlands; 4 Department of Biological Psychology, VU University Amsterdam & Institute for Health and Care Research (EMGO+), VU Medical Center, Amsterdam, The Netherlands; 5 Department of Internal Medicine, Division of Nephrology, University of Groningen, University Medical Center Groningen, Groningen, The Netherlands; Temple University, UNITED STATES

## Abstract

**Objectives:**

In order to investigate the applicability of routine 10s electrocardiogram (ECG) recordings for time-domain heart rate variability (HRV) calculation we explored to what extent these (ultra-)short recordings capture the “actual” HRV.

**Methods:**

The standard deviation of normal-to-normal intervals (SDNN) and the root mean square of successive differences (RMSSD) were measured in 3,387 adults. SDNN and RMSSD were assessed from (ultra)short recordings of 10s(3x), 30s, and 120s and compared to 240s–300s (gold standard) measurements. Pearson’s correlation coefficients (r), Bland-Altman 95% limits of agreement and Cohen’s d statistics were used as agreement analysis techniques.

**Results:**

Agreement between the separate 10s recordings and the 240s-300s recording was already substantial (r = 0.758–0.764/Bias = 0.398–0.416/d = 0.855–0.894 for SDNN; r = 0.853–0.862/Bias = 0.079–0.096/d = 0.150–0.171 for RMSSD), and improved further when three 10s periods were averaged (r = 0.863/Bias = 0.406/d = 0.874 for SDNN; r = 0.941/Bias = 0.088/d = 0.167 for RMSSD). Agreement increased with recording length and reached near perfect agreement at 120s (r = 0.956/Bias = 0.064/d = 0.137 for SDNN; r = 0.986/Bias = 0.014/d = 0.027 for RMSSD). For all recording lengths and agreement measures, RMSSD outperformed SDNN.

**Conclusions:**

Our results confirm that it is unnecessary to use recordings longer than 120s to obtain accurate measures of RMSSD and SDNN in the time domain. Even a single 10s (standard ECG) recording yields a valid RMSSD measurement, although an average over multiple 10s ECGs is preferable. For SDNN we would recommend either 30s or multiple 10s ECGs. Future research projects using time-domain HRV parameters, e.g. genetic epidemiological studies, could calculate HRV from (ultra-)short ECGs enabling such projects to be performed at a large scale.

## Introduction

Heart rate variability (HRV) quantifies beat-to-beat fluctuations in heart rate and is considered an index of cardiac parasympathetic nervous system activity [1–3]. In the general population reduced HRV has been associated with increased risk of coronary heart disease [4], cardiac mortality [5], and all-cause mortality [6].

HRV is calculated from time series of beat-to-beat heart-rate data [3]. For our study we focused on two time-domain HRV measurements: the Standard Deviation of the normal-to-normal intervals (SDNN) and the Root Mean Square of Successive Differences (RMSSD) between adjacent NNs. Both are easy to calculate and among the most widely used indices of HRV [7]. SDNN estimates overall HRV, while RMSSD estimates short-term components of HRV [4]. However in both clinical practice and research, ECGs of 10s or 20s are routinely collected and constitute a vast and potentially valuable resource. Additionally, short-term recordings are suitable for large scale studies, because they impose a minimal burden on the subject and can be made under standardized conditions [8].

Currently it is not known to what extent (ultra-)short ECG recordings of 10s to 20s manage to capture the “actual” HRV of a subject at rest. Only a limited number of studies have specifically investigated the validity [9,10] and reproducibility [8,11] of (ultra-)short HRV measurements. However, these studies had very small sample sizes (n≤70 [10]). In addition these studies used (intra-class) correlation coefficients between two measurements of different recording lengths, but this does not account for neither the potential differences in means between two measurements [12,13] nor communicates the degree of the differences [14].

Given these methodological limitations in the existing literature [9–11] we investigated in a sample of 3,387 subjects to what extent (ultra-)short recordings capture the “actual” HRV. We evaluated recordings of 10s, 30s, and 120s selected from the longest (gold-standard) recording of 240s to 300s. SDNN and RMSSD measured from the (ultra-)short recordings were compared to the gold standard [12,13]. In addition to correlation coefficients we calculated Bland-Altman’s 95% limits of agreement (LoA) and Cohen’s d statistics to evaluate the agreement of SDNN and RMSSD measured from (ultra-)short recordings and the gold standard.

## Methods

### Study population

Our study population consisted of subjects from the “Prevention of Renal and Vascular End-stage Disease” (PREVEND) study, a prospective cohort composed of men and women aged from 44.8 to 63.2 years living in Groningen, The Netherlands [15,16]. It was initiated to investigate the natural course of increased albuminuria levels and its association to renal and cardiovascular disease. PREVEND subjects completed a first survey between 1997–1998. During the second (between 2001–2003) and third (between 2003–2006) screening rounds beat-to-beat blood pressure recordings were collected during a 15minute supine resting period, which were used for HRV calculations (details given below). All subjects gave written informed consent. The PREVEND study was approved by the medical ethics committee of the University Medical Center Groningen and conducted in accordance to the Helsinki Declaration guidelines.

### Measurement procedure

Using a standardized procedure, continuous beat-to-beat pressure recordings on the middle finger using a Portapres® pressure recording device (FMS Finapres Medical systems BV, Amsterdam, The Netherlands) and Beatscope software (Finapres Medical Systems, Amsterdam, The Netherlands) were used to measure NN-interval time series. The cuff of the Portapres® was placed on the middle finger of the dominant arm. The subjects were measured in the supine position in a quiet room at constant temperature (22°C), breathing spontaneously and holding the Portapres cuff at heart level, and were not allowed to talk or move during the measurement.

### Processing of data

**Fig 1:**
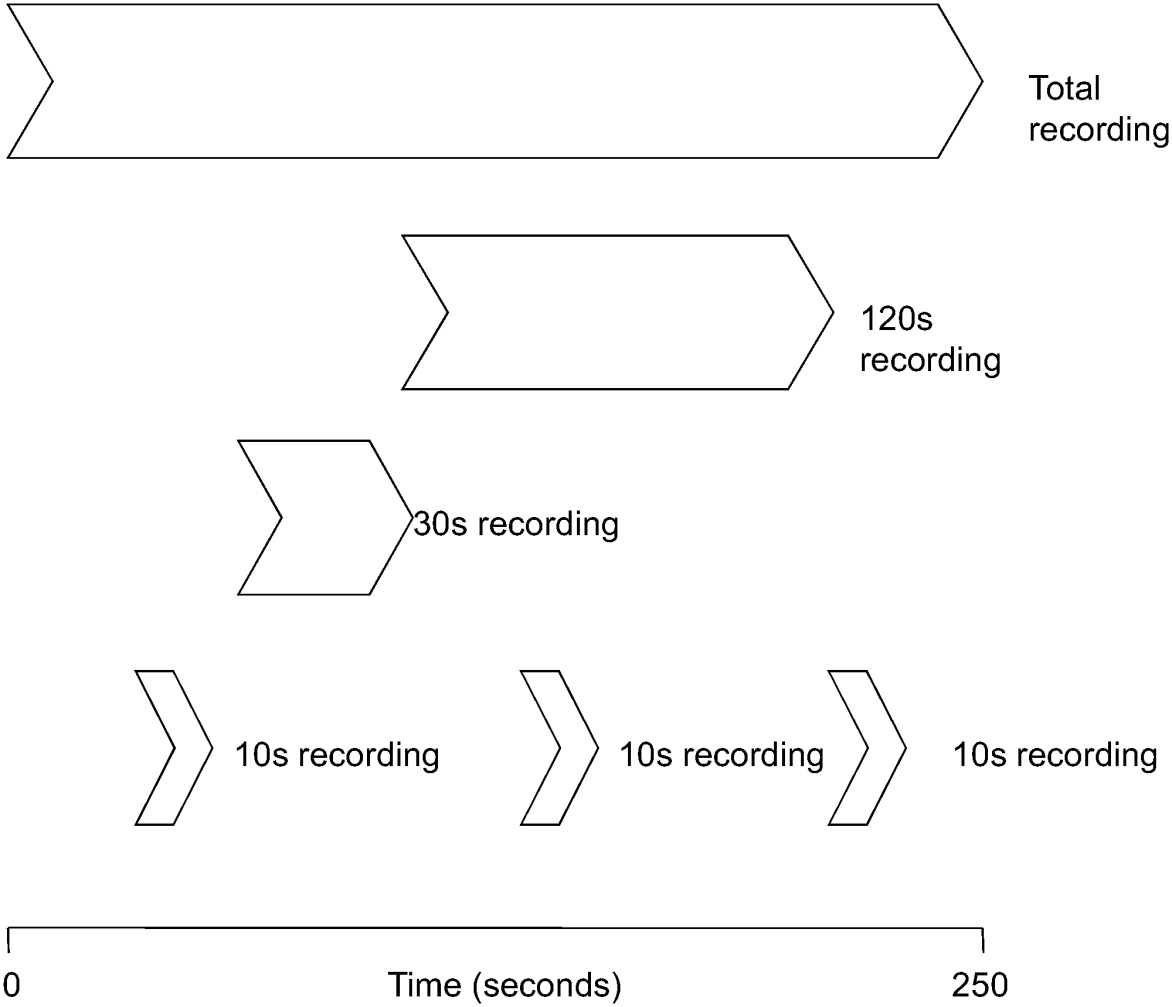
Representation of the 10s, 30s and 120s recordings selected from the total recording period (240s–300s).

Before HRV analysis the pulse wave data was visually pre-processed to exclude non-sinus rhythm, ectopic beats, and artifacts, such as premature ventricular beats, electrical ‘noise’, or aberrant beats. NN-intervals from the beat-to-beat blood pressure signals were detected, with an accuracy ±5ms. Artifacts were removed and the resulting gaps were interpolated. The NN-interval detection and interpolation algorithm used has been previously described [17]. When a recording measured had more than 5% interpolated NN-intervals, the data were considered invalid and discarded. From these processed beat-to-beat blood pressure signals the deflections were detected and all intervals in-between these deflections (NN-intervals) were used to calculate SDNN and RMSSD. SDNN and RMSSD were obtained using the CARSPAN 2.0 program (IECProgramma, Groningen, the Netherlands), which is a software package specifically designed for cardiovascular spectral analysis [18]. From the 15 min of recorded signal we selected the last 4 to 5 min with a stationary time series. This recording length of 240s to 300s of high quality signal was considered the gold-standard recording length. SDNN and RMSSD were calculated for this total recording length. Three non-overlapping 10s recordings were randomly selected from a subject’s total recording, while periods of 30s and 120s were selected from the start of the total recording. In addition we also calculated the average SDNN and RMSSD of the three 10s recordings (Avg10s) (Fig 1). After data processing we had HRV data of 3,387 subjects that were used for analysis.

### Statistical analyses

Prior to the analyses, SDNN and RMSSD data were log-transformed to obtain approximately normal distributions. Pearson’s correlation coefficients(r) for SDNN and RMSSD were calculated between the gold-standard recording and the three separate 10s, the Avg10s, the 30s, and the 120s recordings. However, a correlation coefficient is blind to the possibility of bias caused by the differences in the mean and/or standard deviation (SD) between the two measurements. More specific, a strong correlation does not necessarily imply a close agreement. Therefore the Bland-Altman procedure was used to calculate 95% LoA [12,13]. In contrast to the traditional Bland-Altman plots we plotted the measurement of the gold standard on the x-axis [19]. The bias was calculated as the mean difference between the HRV measurements of the gold standard and those of the (ultra-)short recording periods. Furthermore, we calculated Cohen’s d statistics to quantify the bias of the HRV measurements of different recording lengths relative to their within-group variations [14]. This was done by dividing the bias in HRV by the standard deviation (SD) of the total recording. For example, a Cohen’s d of 0.027 is the difference between two recording means of 2.7% of the SD of the total recording could be interpreted as a very small effect (where d = 0.20 is a small, d = 0.50 is a moderate, and d = 0.80 is a large difference) [14,20]. In addition, to measure the reliability of our 10s recording periods we calculated the intra-class correlation coefficients (ICC; absolute agreement, two-way analysis of variance) between the three 10s measurements for both RMSSD and SDNN. Stata v11.2 (StataCorp LP, Texas, USA) was used for all statistical analysis. P-values <0.05 were considered statistical significant.

### Simulation study

As a result of our study design, measures based on the (ultra-)short segments are not independent from the total (gold standard) period from which they were selected, which automatically generates an inflation of the correlations, Cohen’s ds, and 95% LoAs that we determine in this study. Therefore, we conducted a simulation study using a bootstrapping procedure in order to assess the correlations, 95% LoAs, and Cohen’s d statistics expected under the null hypothesis of no agreement between the measurements of the (ultra-)short recordings and the remainder of the total recording. That is, the only agreement between HRV measurements from the (ultra-)short and total recordings arises from the (ultra-)short recording being part of the total recording.

The HRV values for the remainders of the total recording (i.e. of length 230-290s for the 10s recordings, of length 210-270s for the 30s recordings, and of length 120-180s for the 120s recordings) were approximated by subtracting HRV based on the (ultra-)short recording from HRV of the total recording using a mathematical formula for decomposing variances. Formula (1) shows how HRV from a 290s recording is approximated by subtracting HRV from a 10s recording from a total recording of 300s.

$$HRV(290s)\;=\;\sqrt{\frac{{HRV(300s)}^{2}∙(N(300s)-1)\;-\;{HRV(10s)}^{2}∙(N(10s)-1)}{N(300s)-N(10s)-1}},$$ (1)

where N(*x*s) is the number of NN intervals for the *x*s recording. Next 3,387 HRV values from the actual data set of (ultra-)short recordings and 3,387 HRV values from the actual data set of corresponding remainders were drawn independently of each other with replacement and then each pair of HRV values was combined to approximate HRV from a total recording using a mathematical formula for adding independent SDs. For example, to simulate HRV from a 300s recording under the null hypothesis, HRV from a 10s recording was selected as well as HRV from a 290s recording and from these two values HRV from a 300s recording was approximated using Formula (2).

$$HRV(300s)\;=\;\sqrt{\frac{{HRV(10s)}^{2}∙(N(10s)-1)+{HRV(290s)}^{2}∙(N(290s)-1)}{N(10s)+N(290s)-1}}$$ (2)

**Fig 2:**
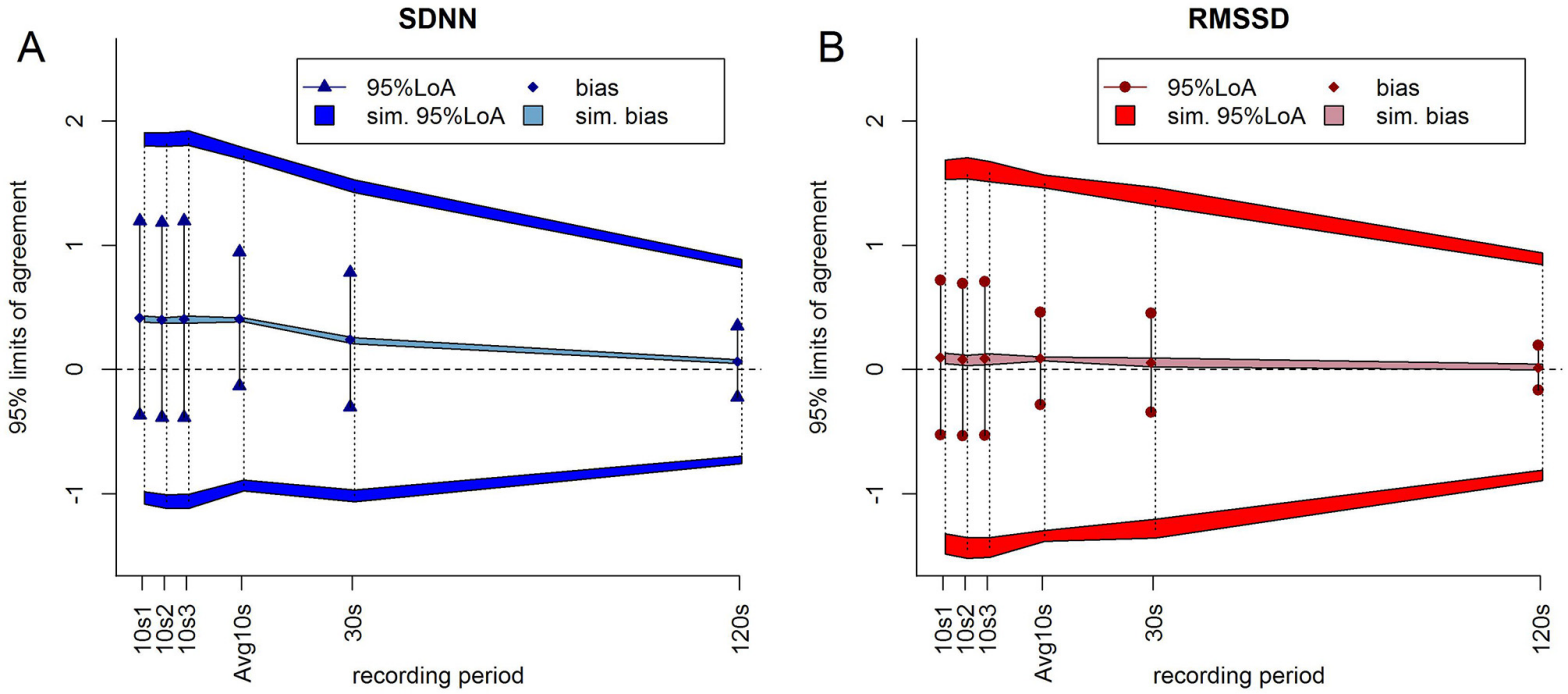
Biases and 95% LoAs milliseconds(ms) of the log-transformed (a) SDNN [in blue] and (b) RMSSD [in red] measured from the recordings with different time lengths compared to the total recording. The diamonds are the observed bias and the lines represent the intervals defined by the observed 95%LoAs. The bands show the 95% reference ranges of the simulated biases and the dark gray bands show the 95% reference ranges of the upper and lower 95%LoAs from the simulation. The dotted lines represent the intervals defined by the simulated 95%LoAs. For both SDNN and RMSSD for all recording lengths the observed biases did not differ from the expectation, but the observed 95%LoAs were much narrower than expected.

Correlation coefficients, 95% LoAs, and Cohen’s ds were computed to determine the agreement of the 10, 30, and 120 NN interval measurements with the total recording under the null hypothesis. This procedure was repeated 1,000 times and for each of the HRV variables (SDNN or RMSSD) measured from each of the (ultra-)short recordings (10s, Avg10s, 30s, and 120s) 95% reference ranges were determined for the correlation coefficients, 95% LoAs, and Cohen’s ds. The observed values were compared to these ranges expecting that the observed values will show more agreement than expected and hence fall outside the simulated reference ranges (see Fig 2). An observed value outside the corresponding reference range indicates a significant difference (p<0.05).

## Results

In our sample of 3,387 subjects the mean age was 53 years and 51% were women. The average total recording length was 294s (min-max:239-302s) with a total population heart rate average of 68(SD:±10) beats per minute. At the second screening, our total population had 6.7% of subjects with a recent cardiovascular event, 39% were hypertensive, 7.6% had diabetes mellitus type 2, 34% had hypercholesterolemia and 22% had chronic kidney disease. Median values for SDNN and RMSSD were similar for males and females (see Table 1). We observed the usual strong inverse correlation with age for both SDNN (r = -0.30) and RMSSD (r = -0.28). The 3,387 subjects used in the current study that had good quality HRV measures available constituted about half of the total sample size of the second screening of PREVEND. As shown in Table 2 characteristics of the subjects used in the current study were very similar to those of subjects not used in the current study. Table 3 shows the raw and natural log transformed SDNN and RMSSD categorized by recording length. It shows that the mean values of RMSSD and, particularly, SDNN increased for longer recording lengths. This increase was 1.32ms for the mean RMSSD (from 28.16 for Avg10s to 29.48 for the total recording), while the mean SDNN increased 9.94ms (from 25.87 for Avg10s to 35.81 for the total recording).

**Table 1:** Demographic characteristics of all subjects included in the current study at the second screening of the PREVEND cohort.

Variable	Total	Males	Females
N	3387 (100%)	1658 (49%)	1729 (51%)
Age, yrs	53 [44.8–63.2]	53.9 [45.2–64.6]	52.8 [44.3–60.9]
Black race, n	26 (0.8%)	10 (0.6%)	16 (0.9%)
Height, cm	172 (9.6)	179 (7.2)	166 (7.0)
Weight, kg	80 (15)	86 (13)	74 (14)
BMI, kg/m^2^	27 (4.5)	27 (3.8)	27 (5.0)
WHR	0.90 (0.084)	0.96 (0.066)	0.85 (0.064)
Recent CV event^a^	106 (3.1%)	72 (4.3%)	34 (2.0%)
Heart rate, beats/min	68 (10)	67 (10)	70 (9.6)
Blood pressure			
SBP, mmHg	127 (19)	131 (18)	123 (19)
DBP, mmHg	74 (9.0)	77 (8.7)	71 (8.5)
MAP, mmHg	93 (12)	96 (12)	90 (12)
Antihypertensive T_x_	729 (22%)	378 (23%)	351 (21%)
Hypertension^b^	1192 (39%)	639 (44%)	553 (35%)
Diabetes			
Fasting glucose, mmol/L	4.8 [4.4–5.3]	5.0 [4.5–5.4]	4.7 [4.4–5.2]
Antidiabetic T_x_	107 (3.2%)	59 (3.7%)	48 (2.8%)
Diabetes Mellitus^c^	222 (7.6%)	116 (8.4%)	106 (6.9%)
Smoking			
Never	960 (28%)	396 (24%)	564 (33%)
Former	1415 (42%)	764 (47%)	651 (38%)
Current			
<6 cigarettes/day	141 (4.2%)	67 (4.1%)	74 (4.3%)
6–20 cigarettes/day	685 (20%)	335 (20.5%)	350 (21%)
>20 cigarettes/day	141 (4.2%)	73 (4.5%)	68 (4.0%)
Lipids			
Total cholesterol, mmol/L	5.5 (1.0)	5.4 (1.0)	5.5 (1.0)
Lipid lowering T_x_	296 (9.0%)	163 (1.01%)	133 (7.8%)
Hypercholesterolemia^d^	1043 (34%)	512 (31%)	531 (31%)
Renal characteristics			
Serum creatinine, mmol/L	0.82 (0.21)	0.91 (0.18)	0.74 (0.20)
Serum cystatin-C, mg/L	0.91 (0.20)	0.94 (0.20)	0.88 (0.20)
eGFR^e^, ml/min/1.73m^2^	92 (17)	92 (17)	91 (16)
UAE, mg/24h	9.0 [6.2–17]	10 [6.8–23]	8.0 [5.8–14]
CKD^f^	692 (22%)	427 (26%)	265 (16%)
HRV			
SDNN, ms	31.9 [23.8–43.6]	32.0 [23.3–45.1]	31.9 [24.1–42.3]
RMSSD, ms	24.5 [17.1–35.1]	23.4 [16.6–33.8]	25.8 [17.9–36.6]

Data are expressed as mean (sd), number (%), or median [IQR] in case of skewed distributions. BMI body mass index; WHR waist-hip ratio; CV cardiovascular; SBP systolic blood pressure; DBP diastolic blood pressure; MAP mean arterial pressure; T_x_ therapy; LDL low density lipoprotein; eGFR estimated glomerular filtration rate; UAE urinary albumin excretion; CKD chronic kidney disease, HRV heart rate variability; SDNN standard deviation of normal-to-normal RR-intervals; RMSSD; root mean square of successive differences of adjacent RR-intervals.

^a^ Defined as a clinical diagnosis of any of the following: acute myocardial infarction, (sub)acute ischemic heart disease, coronary artery bypass grafting, percutaneous transluminal coronary angioplasty, subarachnoid haemorrhage, intracerebral haemorrhage, occlusion/stenosis of the (pre)cerebral arteries, and other vascular interventions such as percutaneous transluminal angioplasty or bypass grafting of aorta/peripheral vessels.

^***b***^ Defined as SBP ≥ 140mmHg, DBP ≥ 90mmHg, or antihypertensive T_x_.

^***c***^ Defined as fasting glucose > 7 mmol/L, or antidiabetic T_x_. All cases were type 2 diabetes.

^***d***^ Defined as total cholesterol ≥ 6.21 mmol/L, or lipid lowering T_x_.

^***e***^ Calculated using the CKD-EPI serum creatinine—serum cystatin C equation.

^***f***^ Defined as either eGFR < 60ml/min/1.73m^2^ or UAE ≥ 30mg/24h.

Smoking was self-reported “never”, “former”, “currently <6”, currently “6–20” or “currently >20”. T_x_ was reported “yes” “no” or “unknown”.

**Table 2:** Demographic characteristics of subjects from the PREVEND cohort that were included in and excluded from our study.

**N**	6894 (100%)	3387 (49.1%)	3507 (50.9%)	n/a
**Age, yrs**	53 [44.0–63.6]	53 [44.8–63.2]	52.0 [43.2–63.8]	0.020*
**Male**	3444 (50%)	1658 (49.0%)	1786 (50.9%)	0.101
**Black race**	64 (0.9%)	26 (0.8%)	38 (1.1%)	0.172
**Height, cm**	173 (9.5)	172 (9.6)	173 (9.5)	0.033*
**Weight, kg**	79.9 (14.6)	80.0 (14.7)	79.8 (14.5)	0.470
**BMI, kg/m^2^**	26.8 (4.4)	26.9 (4.5)	26.6 (4.3)	0.020*
**WHR**	0.90 (0.086)	0.90 (0.084)	0.90 (0.087)	0.032*
**Recent CV event^a^**	218 (3.2%)	106 (3.1%)	112 (3.2%)	0.879
**Heart rate, beats/min**	68.5 (10.1)	68.4 (10.1)	68.5 (10.2)	0.933
**Blood pressure**				
**SBP, mmHg**	126 (19)	127 (19)	126 (19)	0.264
**DBP, mmHg**	73 (9.1)	74 (9.0)	73 (9.2)	0.050*
**MAP, mmHg**	93 (12)	93 (12)	92 (12)	0.230
**Antihypertensive T_x_**	1414 (20.8%)	729 (21.5%)	685 (19.5%)	0.066
**Hypertension^b^**	2380 (38.6%)	1192 (39.1%)	1188 (38.2%)	0.452
**Diabetes**				
**Fasting glucose, mmol/L**	4.8 [4.4–5.3]	4.8 [4.4–5.3]	4.8 [4.4–5.3]	0.358
**Antidiabetic T_x_**	235 (3.5%)	107 (3.2%)	128 (3.7%)	0.166
**Diabetes Mellitus^c^**	454 (7.7%)	222 (7.6%)	232 (7.8%)	0.810
**Smoking**				0.235
**Never**	1969 (28.9%)	960 (28.7%)	1009 (29.1%)	
**Former**	2922 (42.9%)	1415 (42.3%)	1507 (43.5%)	
**Current**				
** <6 cigarettes/day**	307 (4.5%)	141 (4.2%)	166 (4.8%)	
** 6–20 cigarettes/day**	1346 (19.8%)	685 (20.4%)	661 (19.1%)	
** >20 cigarettes/day**	264 (3.9%)	141 (4.2%)	123 (3.5%)	
**Lipids**				
**Total cholesterol, mmol/L**	5.4 (1.1)	5.5 (1.0)	5.4 (1.1)	0.001*
**Lipid lowering T_x_**	592 (8.8%)	296 (9.0%)	296 (8.7%)	0.335
**Hypercholesterolemia^d^**	2055 (33.4%)	1043 (34.4%)	1012 (32.5%)	0.127
**Renal characteristics**				
**Serum creatinine, mmol/L**	0.83 (0.23)	0.82 (0.21)	0.83 (0.25)	0.218
**Serum cystatin-C, mg/L**	0.91 (0.21)	0.91 (0.20)	0.91 (0.22)	0.469
**eGFR^e^, ml/min/1.73m^2^**	92 (17)	92 (17)	92 (18)	0.563
**UAE, mg/24h**	8.8 [6.1–16.5]	9.0 [6.2–17]	8.7 [6.0–16]	0.076
**CKD^f^**	1391 (20%)	692 (21.5%)	699 (21.2%)	0.746

Data are expressed as mean (sd), number (%), or median [IQR] in case of skewed distributions. BMI body mass index; WHR waist-hip ratio; CV cardiovascular; SBP systolic blood pressure; DBP diastolic blood pressure; MAP mean arterial pressure; T_x_ therapy; LDL low density lipoprotein; eGFR estimated glomerular filtration rate; UAE urinary albumin excretion; CKD chronic kidney disease;

^***a***^ Defined as a clinical diagnosis of any of the following: acute myocardial infarction, (sub)acute ischemic heart disease, coronary artery bypass grafting, percutaneous transluminal coronary angioplasty, subarachnoid haemorrhage, intracerebral haemorrhage, occlusion/stenosis of the (pre)cerebral arteries, and other vascular interventions such as percutaneous transluminal angioplasty or bypass grafting of aorta/peripheral vessels.

^***b***^ Defined as SBP ≥ 140mmHg, DBP ≥ 90mmHg, or antihypertensive T_x_.

^***c***^ Defined as fasting glucose > 7 mmol/L, or antidiabetic T_x_. All cases were type 2 diabetes.

^***d***^ Defined as total cholesterol ≥ 6.21 mmol/L, or lipid lowering T_x_.

^***e***^ Calculated using the CKD-EPI serum creatinine—serum cystatin C equation.

^***f***^ Defined as either eGFR < 60ml/min/1.73m^2^ or UAE ≥ 30mg/24h.

Smoking was self-reported “never”, “former”, “currently <6”, currently “6–20” or “currently >20”. T_x_ was reported “yes” “no” or “unknown”.

Two-sided p-values were calculated using t-tests, Mann-Whitney U-tests, and χ^2^-tests where applicable.

* shows statistically significant: p-value ≤ 0.05

**Table 3:** Descriptive statistics of raw and natural log transformed SDNN and RMSSD of 3,387 individuals categorized by recording period.

Recording	SDNN mean (SD)	lnSDNN mean (SD)	RMSSD mean (SD)	lnRMSSD mean (SD)
**10s recording 1**	25.48 (18.35)	3.05 (0.60)	27.78 (23.27)	3.13 (0.59)
**10s recording 2**	26.18 (19.99)	3.07 (0.61)	28.57 (25.92)	3.15 (0.61)
**10s recording 3**	25.95 (19.04)	3.06 (0.61)	28.16 (23.78)	3.14 (0.60)
**Avg10s**	25.87 (16.86)	3.06 (0.53)	28.17 (22.17)	3.14 (0.55)
**30s recording**	29.14 (17.87)	3.23 (0.53)	28.22 (21.99)	3.17 (0.55)
**120s recording**	33.96 (18.64)	3.40 (0.49)	29.15 (22.43)	3.21 (0.53)
**total recording**	35.81 (18.57)	3.47 (0.46)	29.48 (22.51)	3.23 (0.53)

s: seconds; SDNN: standard deviation of normal-to-normal intervals in milliseconds; RMSSD: root mean square of successive differences in milliseconds; lnSDNN: natural logarithm of SDNN; lnRMSSD: natural logarithm of RMSSD; SD: Standard Deviation; Avg10s: the average of the measurements from the three 10s recordings.

### Pearson’s correlation coefficients

**Fig 3:**
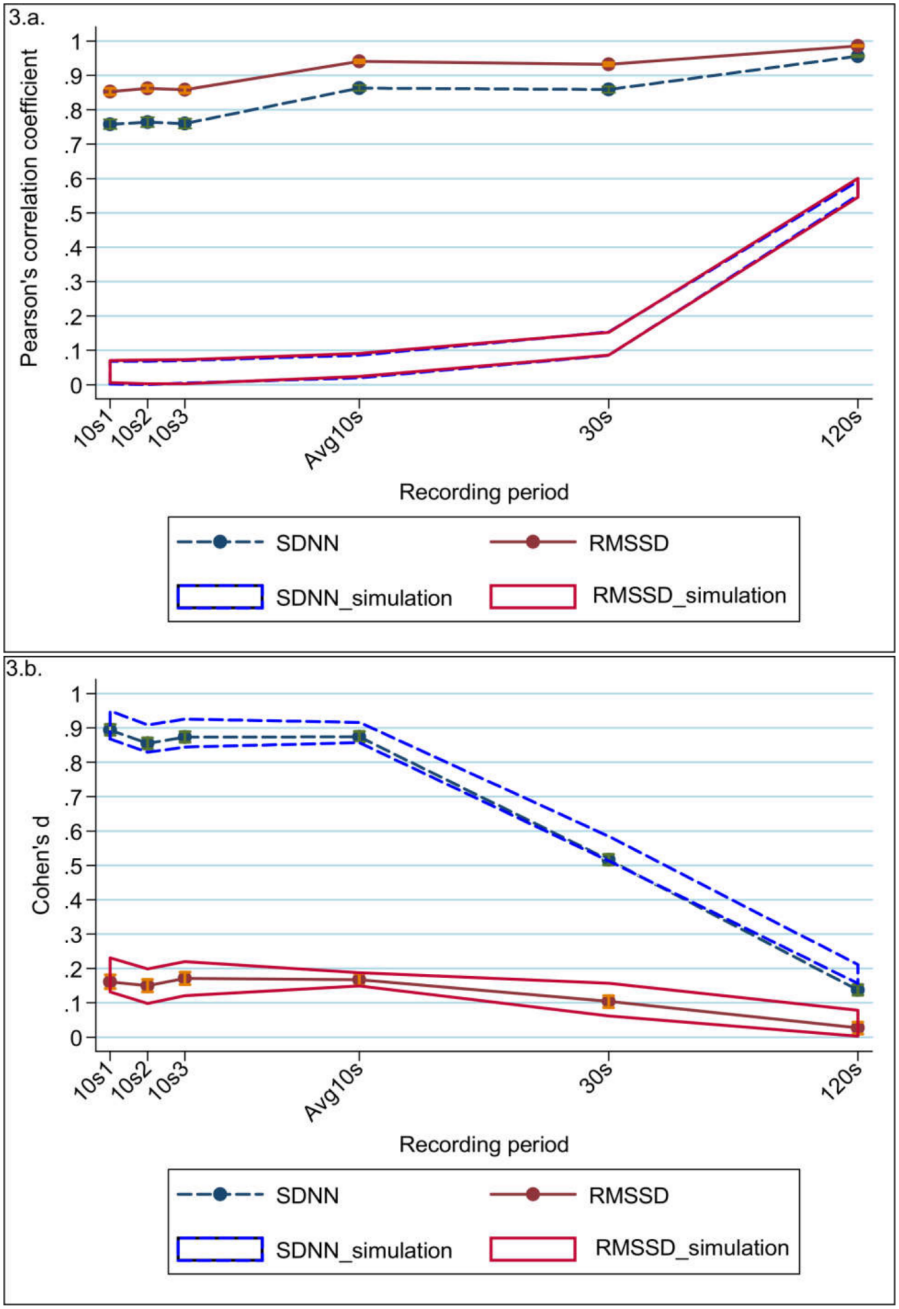
Agreement statistics of the natural logarithm of SDNN (blue dashed line) and RMSSD (red solid line) measured from the recordings with different time lengths compared to the total recording. Agreement is measured using (a) Pearson’s correlation coefficient, and (b) Cohen’s d statistic. The error bars (orange for RMSSD; green for SDNN) represent 95% confidence intervals of the mean. The red solid line band (representing RMSSD) and the blue dashed line band (representing SDNN) are the expected values under the null hypothesis as a result of our simulation analysis. For both SDNN and RMSSD for all recording lengths the observed correlations were considerably larger than expected under the null hypothesis, but the Cohen’s ds for both SDNN and RMSSD did not differ from the expectation.

Correlation between a single 10s recording and the gold-standard recording was already substantial (r = 0.758–0.764 for SDNN; r = 0.853–0.862 for RMSSD) and increased significantly for Avg10s (r = 0.863 for SDNN; r = 0.941 for RMSSD) [Table 4; Fig 3a]. For both SDNN and RMSSD the correlations of Avg10s were similar to those of the 30s recordings (r = 0.863 and 0.859, respectively for SDNN; r = 0.941 and 0.932, respectively for RMSSD). Near perfect correlations with the gold standard were found for the measurements of the 120s recording (r = 0.956 for SDNN and r = 0.986 for RMSSD). Overall the correlations were lower for SDNN compared to RMSSD, but this difference became smaller with the increase of recording length. The differences in correlation between SDNN and RMSSD were significant as shown by their non-overlapping 95%CI.

**Table 4:** Agreement statistics of the natural logarithm of SDNN and RMSSD for the different recordings compared to the gold standard.

	SDNN				RMSSD			
	Pearson’s correlation (95% CI)	Bias (95% CI)	95% LoA	Cohen’s d (95% CI)	Pearson’s correlation (95% CI)	Bias (95% CI)	95% LoA	Cohen’s d statistics (95% CI)
**10s recording1**	0.758 (0.744–0.772)	0.416 (0.403–0.429)	-0.362–1.193	0.894 (0.879–0.910)	0.853 (0.843–0.862)	0.096 (0.085–0.106)	-0.525–0.716	0.161 (0.141–0.181)
**10s recording2**	0.764 (0.750–0.778)	0.398 (0.385–0.411)	-0.386–1.181	0.855 (0.839–0.871)	0.862 (0.854–0.871)	0.079 (0.069–0.089)	-0.533–0.691	0.150 (0.132–0.181)
**10s recording3**	0.760 (0.746–0.774)	0.406 (0.393–0.419)	-0.386–1.198	0.873 (0.858–0.889)	0.858 (0.849–0.866)	0.090 (0.080–0.101)	-0.528–0.709	0.171 (0.130–0.170)
**Avg10s**	0.863 (0.855–0.872)	0.406 (0.397–0.416)	-0.131–0.944	0.874 (0.859–0.890)	0.941 (0.937–0.945)	0.088 (0.082–0.095)	-0.282–0.459	0.167 (0.153–0.189)
**30s recording**	0.859 (0.850–0.868)	0.240 (0.231–0.249)	-0.300–0.780	0.516 (0.501–0.532)	0.932 (0.928–0.937)	0.055 (0.048–0.061)	-0.343–0.453	0.104 (0.086–0.121)
**120s recording**	0.956 (0.953–0.959)	0.064 (0.059–0.069)	-0.222–0.349	0.137 (0.122–0.153)	0.986 (0.985–0.987)	0.014 (0.011–0.017)	-0.166–0.194	0.027 (0.009–0.044)

CI: confidence interval; LoA: limits of agreement; s: seconds; Avg10s: the average of the measurements from the three 10s recordings

### Bland-Altman plots

**Fig 4:**
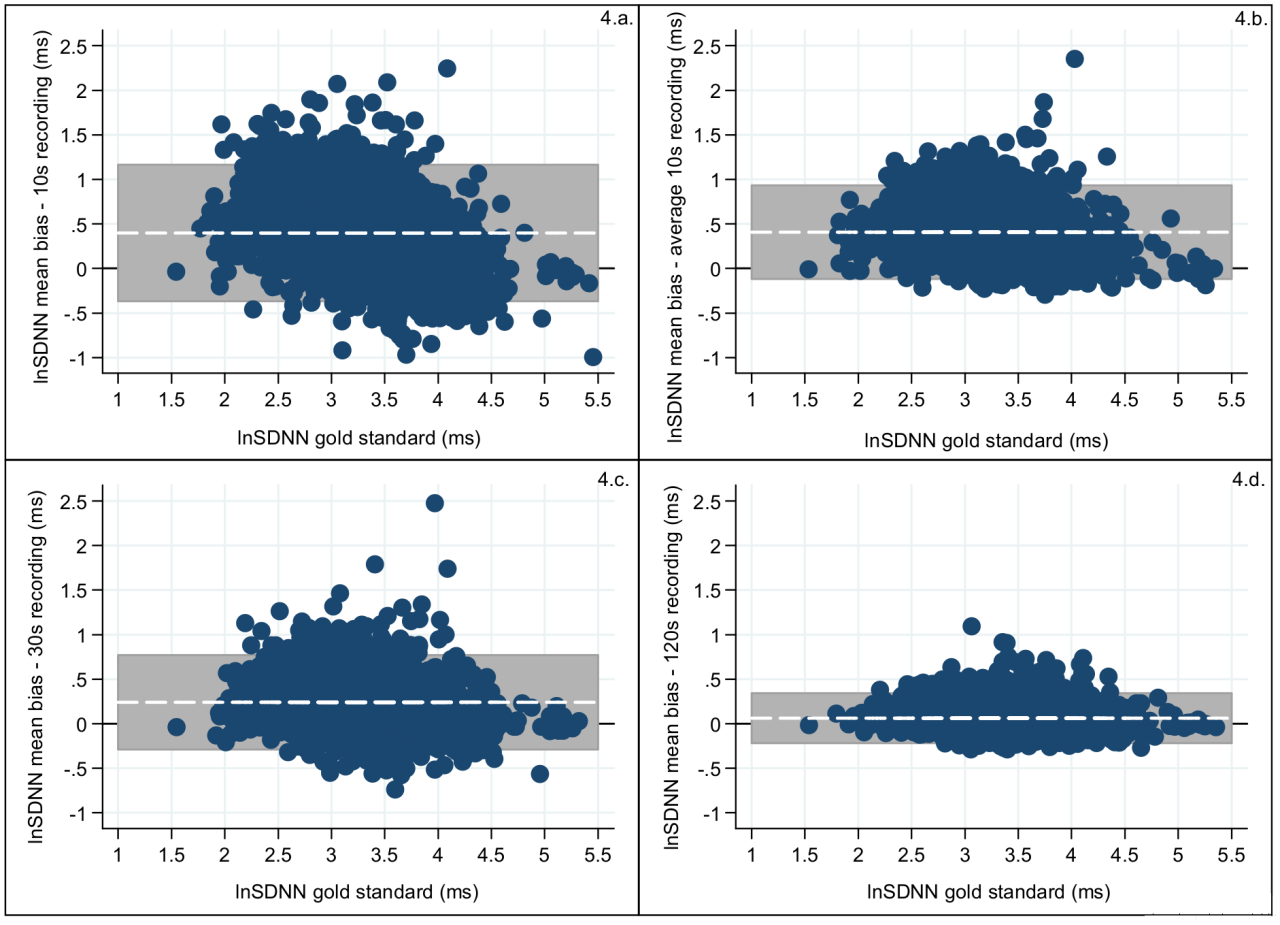
Measurement error of the log transformed values for SDNN (blue dots) calculated as the difference between the total recording and: (a) a 10s recording; (b) the average of the three 10s recording; (c) the 30s recording; (d) the 120s recording. The x-axis is the log transformed SDNN of the total block and the y-axis is the bias of the (ultra-)short recording for log transformed SDNN compared to the gold standard. The grey shaded area represents the interval between the 95%LoA and the white line represents the bias. The black horizontal line is the reference line of no bias (y = 0).

**Fig 5:**
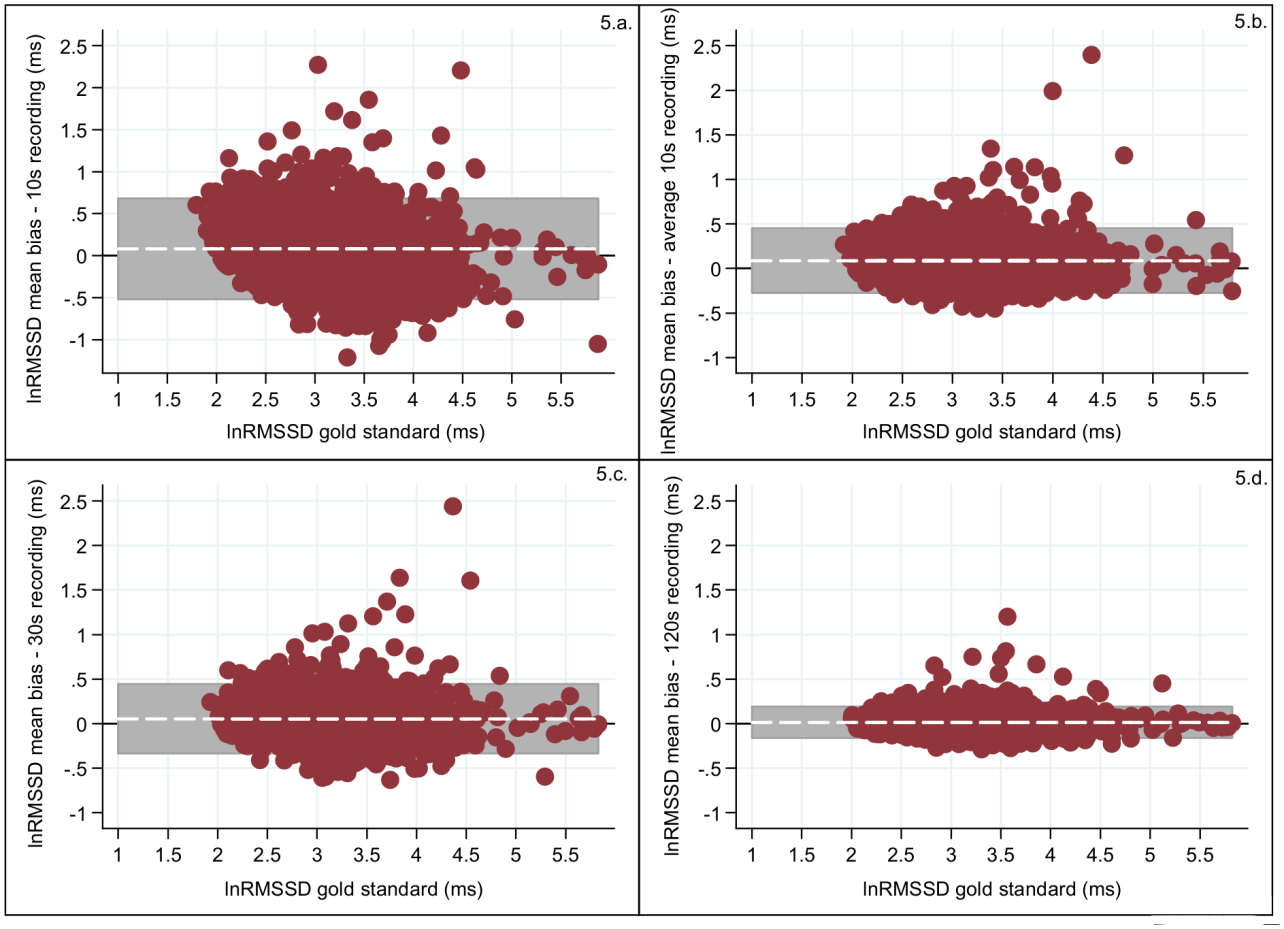
Measurement error of the log transformed values for RMSSD (red dots) calculated as the difference between the total recording and: (a) a 10s recording; (b) the average of the three 10s recording; (c) the 30s recording; (d) the 120s recording. The x-axis is the log transformed RMSSD of the total block and the y-axis is the bias of the (ultra-)short recording for log transformed RMSSD compared to the gold standard. The grey shaded area represents the interval between the 95%LoA and the white line represents the bias. The horizontal black line is the reference line of no bias (y = 0).

Decrease in the bias and in the width of the 95%LoA interval was observed as the recording length increased (Figs 4 and 5; Table 4) for both SDNN and RMSSD. The three 10s recording periods revealed similar biases (for SDNN 0.398–0.416 and for RMSSD 0.079–0.096) and also the 95%LoAs for the three 10s period were similar for both SDNN (widest 95%LoA = -0.386–1.198) and RMSSD (widest 95%LoA = -0.525–0.716). A slight increase in bias was observed for Avg10s (SDNN:0.406 and RMSSD:0.088), but the 95%LoA for both SDNN (95%LoA = -0.131–0.944) and RMSSD (95%LoA = -0.282–0.459) became narrower. For RMSSD the 95%LoA for the measurements from the 30s recordings were equally wide as for Avg10s and the biases for RMSSD (30s:0.055; 95%LoA = -0.343–0.453) were also similar, but for SDNN the 30s recordings both the bias and 95%LoA improved substantially (0.240; 95%LoA = -0.300–0.780) compared to those from the Avg10s recording. An almost negligible bias was found for the 120s recordings for both HRV traits, where SDNN had a bias of 0.064 (95%LoA = -0.059–0.069) and RMSSD of 0.014 (95%LoA = -0.011–0.017) [Table 4]. Overall the biases and the intervals defined by the 95%LoA were smaller for RMSSD compared to SDNN.

### Cohen’s d statistics

For the three 10s recording periods compared to the gold standard Cohen’s d was small for RMSSD (d = 0.150–0.171), but large for SDNN (d = 0.855–0.894) (Table 4; Fig 3b). For the Avg10s recordings, similar Cohen’s d were found (SDNN: d = 0.874; RMSSD: d = 0.167). Cohen’s d for the 30s recording periods compared to the gold standard was moderate for SDNN (d = 0.516) and small for RMSSD (d = 0.104) and it became even smaller for the measurements from the 120s recordings. For SDNN Cohen’s d showed a small difference (d = 0.137) and for RMSSD it was almost negligible (d = 0.027).

### Intra-class correlation coefficients

To measure the reliability of our three 10s recording periods we calculated their ICCs for RMSSD and SDNN (Table 5). The ICC was modest between the three 10s recordings for SDNN (0.657–0.670) and improved for RMSSD (0.740–0.751).

**Table 5:** Interclass correlation coefficients for SDNN and RMSSD between the three different 10s recordings.

	10s recording1 vs. 10s recording2 (95% CI)	10s recording1 vs. 10s recording3 (95% CI)	10s recording2 vs. 10s recording3 (95% CI)
SDNN	0.657 (0.638–0.676)	0.668 (0.649–0.687)	0.670 (0.651–0.689)
RMSSD	0.740 (0.725–0.756)	0.751 (0.736–0.766)	0.746 (0.731–0.761)

CI: confidence interval

## Discussion

In order to investigate the utility of routine 10s ECG recordings for HRV calculation in large-scale epidemiologic studies we evaluated the agreement of SDNN and RMSSD between (ultra-)short recordings and a gold-standard recording of 240s to 300s in 3,387 adults. We showed that RMSSD consistently outperformed SDNN. RMSSD measured from recordings of only 10s in length are already reliable and good proxies for those measured from longer recording lengths (240s-300s), in particular when the measurements from multiple 10s recordings are averaged. For SDNN the measurements from 10s recordings were reliable, but although they correlated moderately (for the single recordings) to strongly (for Avg10s) with the gold standard, agreement was poor in both cases (i.e. Cohen’s d close to 1) and hence are bad proxies. For SDNN measured from 30s recordings the agreement with the gold standard was still only moderate, but sufficient to yield reliable estimates of “actual” SDNN. SDNN and RMSSD measured from 120s recordings were both in high agreement with the gold-standard recordings.

Our findings that RMSSD measured from 10s recordings is a good proxy for the “actual” RMSSD, but that this doesn’t hold for SDNN, are in line with previous studies [8–11]. All of these also observed that measurements from ultra-short recordings yield good estimates of RMSSD, while for SDNN the agreement is not sufficient to provide reliable estimates for the “actual” SDNN. In addition we and others observed that the correlation or agreement increased with an increase of the recording length for RMSSD and especially for SDNN [10,11]. The high dependence of SDNN on recording length is to be expected because SDNN reflects the total power of all HRV frequency components combined whereas RMSSD is a reflection of high frequency HRV components only [3]. Furthermore in line with our findings others have shown that averaging HRV measures obtained from sequential time periods reduces the error imposed by the analysis of very short segments [8,11]. We found that the reliability of the three individual 10s recording periods was substantial, in particular for RMSSD.

In our study we chose to extract the (ultra-)short recordings from the total recording length to specifically address our research question whether HRV measured from (ultra-)short recordings reflect the “actual” HRV. Our design differs from that of Schroeder and colleagues [11], who measured HRV at sequential time periods. Their design is more suited to assess the *repeatability* (or *reliability*) of HRV measurements, while our study design reflects our focus on the *validity* of (ultra-)short recordings for HRV measurements in the time domain (SDNN, RMSSD) compared to a gold-standard recording period of 240s to 300s. A consequence of our study design is that the measurements of the (ultra-)short recordings are not independent of the total recording and hence correlations and agreement measures are expected to be inflated. Nevertheless for both HRV measures all observed correlations were significantly higher and all 95%LoAs significantly smaller than those simulated under the null hypothesis supporting the validity of HRV measurements based on (ultra-)short recordings. The biases and Cohen’s d for both HRV measures did not differ from the expectation. This can be explained by the fact that the distributions of the simulated HRV measures from the (ultra-)short and total recordings are similar to those of the observed ones, leading to similar *mean* differences between the HRV measurements of the gold standard and those of the different (ultra-)short recording periods. However, the *variation* in those paired differences between the observed measurements of the (ultra-)short segments and those of the total recording is smaller than from the respective paired differences of the simulated measurements, explaining the much higher correlation and tighter 95%LoAs.

In this study we analyzed a general population in which the mean age was 53 years and both sexes were included [15,16]. Previous studies [8–11] only included healthy individuals and Dekker et al. [8] further limited their study population to young men (mean ± SD age 25.9±3.8years), thereby reducing the generalizability of their results even more. Therefore our results are more representative of the general population. However, 10s ECGs in cases with cardiac arrhythmias should be used with caution because given the very low number of beats in 10s, one artefact caused by cardiac arrhythmia will make up about 5% of the total duration of the recording depending on the heart rate. Therefore for calculating RMSSD and SDNN we suggest the following criteria: (a) one artefact (of any kind such as detection failure or arrhythmia, harmless or not) at the beginning or at the end of a recording should be excluded and the remaining part of the segment should be used, and (b) other artefacts, not at the beginning or at the end, or more than one, means the exclusion of the entire segment. This is because we would need a continuous segment to calculate the successive differences (i.e. SDNN and RMSSD) and one interruption would imply a great loss of successive differences.

A major strength of our study was the large sample size of 3,387 subjects, which allowed for precise estimates of agreement measures between different recording periods. Furthermore the significance of our study is reinforced by our statistical approach. We calculated not only Pearson’s correlation coefficients to measure the strength of linear association between the recordings, but also used Bland-Altman’s statistics [12,13] and Cohen’s d [14] to evaluate the degree of bias. As pointed out by Altman and Bland correlation coefficients are not sufficient to demonstrate the agreement of measurements [12, 13]. No previous studies have used these different agreement analysis techniques. The importance of considering measurements of differences is demonstrated when comparing our results of the Pearson’s correlation coefficients and Cohen’s d statistics. For instance a substantial decrease in Cohen’s d statistic from Avg10s to 30s is shown for SDNN, while the Pearson’s correlation coefficients remains the same. Therefore, only considering Pearson’s correlation coefficient results for SDNN would lead to an erroneous interpretation.

Unlike other studies [8–11] that also measured frequency domain HRV parameters such as the high frequency (HF) component we limited our study to time domain parameters RMSSD and SDNN. This was because ECGs of less than 60s duration are not sufficient to assess the HF components and ECGs of at least 120s should be used to address the low frequency components [1–3]. Therefore our conclusions do not apply to HRV parameters in the frequency domain.

An important implication of our study is that 10s ECG recordings could be used for calculating time-domain HRV parameters, particularly RMSSD, in future epidemiologic studies. In standard in-clinic evaluation of heart rate dynamics, 300s is the recommended length of measurement [3]. Nevertheless, 10s recordings from 12-lead ECGs are already commonly used to detect resting abnormalities in interval lengths, wave morphology and segment elevation/depressions [10] and have already shown their usefulness as diagnostic tool [5,6,21]. For example, reduced HRV measured from three 10s ECG recordings was recently found to be associated with an increased incidence of heart failure [21]. An example of our findings applicability is genome-wide association studies (GWAS), where large sample sizes are needed to detect small effects of genetic variants. A large number of cohorts may have short ECG recordings available but may not (yet) have measured RMSSD (and SDNN). The increase in sample size when using RMSSD (and SDNN) from these cohorts in a GWAS will most likely outweigh the loss in accuracy of the phenotype measurements and hence permit the identification of more genetic variants.

In summary, from our unprecedented large sample size, the selection of our (ultra-)short recording from our total recording, our careful data processing and our sophisticated statistical analysis we can conclude that particularly RMSSD from (ultra-)short recordings manages to capture HRV well. Even a single 10s (standard) ECG recording yields a valid RMSSD measurement, although averaging over multiple 10s ECGs is preferable. For SDNN we would recommend recordings of at least 30s or, if not available, multiple 10s ECGs. In addition, our study suggests that it is unnecessary to use recordings longer than 120s to obtain accurate measures of RMSSD and SDNN.
